# Using the Yeast Three-Hybrid System to Identify Proteins that Interact with a Phloem-Mobile mRNA

**DOI:** 10.3389/fpls.2012.00189

**Published:** 2012-08-27

**Authors:** Sung Ki Cho, Il-Ho Kang, Tyrell Carr, David J. Hannapel

**Affiliations:** ^1^Plant Biology Major, Iowa State UniversityAmes, IA, USA; ^2^DuPont Company, Crop Protection, Stine-Haskell Research CenterNewark, DE, USA; ^3^Department of Biology and Physical Sciences, Chowan UniversityMurfreesboro, NC, USA

**Keywords:** mobile RNA, potato, *Solanum tuberosum*, yeast three-hybrid, StBEL5, BEL1 family

## Abstract

Heterografting and RNA transport experiments have demonstrated the long-distance mobility of *StBEL5* RNA, its role in controlling tuber formation, and the function of the 503-nt 3′ untranslated region (UTR) of the RNA in mediating transport. Because the 3′ UTR of *StBEL5* is a key element in regulating several aspects of RNA metabolism, a potato leaf cDNA library was screened using the 3′ UTR of *StBEL5* as bait in the yeast three-hybrid (Y3H) system to identify putative partner RNA-binding proteins (RBPs). From this screen, 116 positive cDNA clones were isolated based on nutrient selection, *HIS3* activation, and *lacZ* induction and were sequenced and classified. Thirty-five proteins that were predicted to function in either RNA- or DNA-binding were selected from this pool. Seven were monitored for their expression profiles and further evaluated for their capacity to bind to the 3′ UTR of *StBEL5* using β-galactosidase assays in the Y3H system and RNA gel-shift assays. Among the final selections were two RBPs, a zinc finger protein, and one protein, StLSH10, from a family involved in light signaling. In this study, the Y3H system is presented as a valuable tool to screen and verify interactions between target RNAs and putative RBPs. These results can shed light on the dynamics and composition of plant RNA-protein complexes that function to regulate RNA metabolism.

## Introduction

For plant development, phloem plays important roles in not only transporting nutrients, but also as a conduit for moving signal RNAs and proteins. Full-length, phloem-mobile mRNAs function to integrate environmental cues for plant development via this long-distance signaling pathway (Haywood et al., 2005; Banerjee et al., 2006). There are three types of mobile RNAs in plants; (1) pathogenic viral and viroid RNAs, (2) small RNAs including siRNAs and microRNAs, and (3) full-length cellular RNA transcripts (Kehr and Buhtz, 2008). Although many full-length transcripts have been identified in the phloem, only a few of these transcripts have been confirmed to be mobile through the phloem translocation stream. The best examples of mobile RNAs are *StBEL5* (Banerjee et al., 2006), *CmGAI* (Haywood et al., 2005), and the *Arabidopsis FLOWERING LOCUS T* (Li et al., 2011; Lu et al., 2012). StBEL5 is a transcription factor that works in tandem with Knotted1-types to regulate plant growth (Chen et al., 2003, 2004). RNA detection methods and heterografting experiments demonstrated that *StBEL5* transcripts are present in phloem cells and move across a graft union to localize in stolon tips, the site of tuber induction (Banerjee et al., 2006). This movement of RNA originates in leaf veins and petioles and is induced by a short-day photoperiod, regulated by the untranslated regions, and correlated with enhanced tuber production (Banerjee et al., 2006, 2009). Long-distance movement of the RNA of *GA INSENSITIVE* (*GAI*) has also been clearly established in both cucumber and pumpkin (Haywood et al., 2005; Ham et al., 2009). Recent results suggest that in addition to FT protein, *FT* RNA may also be moving to shoot apices to contribute to systemic floral signaling (Li et al., 2011; Lu et al., 2012).

In general, RNA molecules are associated with RNA-binding proteins (RBPs) in the cell, and a number of RNA-protein interactions have been established. RBPs function in splicing, nuclear export, RNA transport and localization, translation, and stability (Dreyfuss et al., 2002; Fedoroff, 2002). RBPs are involved in coordinating gene expression and also influence the localization of protein synthesis (Lunde et al., 2007). For example, a polypyrimidine-tract binding protein (PTB), designated as CmRBP50, was reported as the core protein of a phloem-mobile ribonucleoprotein complex consisting of six RNAs, including *CmGAI* RNA, and 16 proteins in pumpkin phloem sap (Ham et al., 2009). Commonly, it is the UTRs that function via protein interactions in facilitating the cellular localization of a transcript (Jansen, 2001), in mediating its stability (Lee and Jeong, 2006), or in regulating the efficiency of translation (Barreau et al., 2006). Binding motifs have been identified in the RNAs of animals that function in recognizing RBPs (for review, see Jansen, 2001). These motifs are most predominant in the 3′ UTR (Saunders and Cohen, 1999; Corral-Debrinski et al., 2000; Thio et al., 2000). There are numerous examples demonstrating the importance of the 3′ UTRs in recognizing RBPs that regulate metabolism and movement (Ferrandon et al., 1994; Padmanabhan and Richter, 2006; Irion and St. Johnston, 2007). As a prime example in plants, the 3′ UTR of *StBEL5* plays a significant role in mediating its long-distance transport, controlling translation, and regulating stability (Banerjee et al., 2006, 2009), suggesting the presence of *cis*-elements in this UTR that are recognized by RNA-binding partners.

Although there are several useful biochemical approaches to analyze RNA-protein interactions, the yeast three-hybrid (Y3H) system (Sengupta et al., 1996; Hook et al., 2005) represents a simple but powerful tool for searching a large collection of cDNAs to identify proteins that bind a specific RNA of interest (Cassiday and Maher III, 2003; Gonsalvez et al., 2003; Maniataki et al., 2003; Moore et al., 2003; Campalans et al., 2004; Hwang et al., 2005). Not only does it allow the identification of RNA-protein binding partners but also the dissection of higher-order RNA-protein complexes (Bernstein et al., 2002). Using the 503-nt 3′ UTR of *StBEL5* as bait, the Y3H system was used with a potato leaf cDNA library for screening binding partners that may be involved in the metabolism of the full-length, mobile RNA, *StBEL5*. Initially, more than 100 cDNA clones were isolated from the screening based on nutrient selection and *HIS3* and β-galactosidase activation. Seven proteins were selected based on their putative RNA-binding properties for further analyses and RNA gel-shift assays. These results clearly demonstrate the utility of the Y3H system in identifying candidate RBPs.

## Materials and Methods

### Constructs for the Y3H system

The DNA fragments encoding full-length and truncated forms (D1, T2, and UA baits) of the 3′ UTR of *StBEL5* were amplified with gene-specific primer sets (Table A5 in Appendix) and cloned into pIIIA/MS2-1. The plasmids containing the hybrid RNA fused to the full-length 3′ UTR and the truncated forms were transformed into the YBZ-1 yeast strain. For screening, an amplified leaf cDNA library from potato (*Solanum tuberosum* cv Désirée) similar in design to the stolon library described by Chen et al. (2003) was used. This library was directionally cloned into pAD-GAL4-2.1 (Stratagene, La Jolla, CA, USA) and was a generous gift from Salomé Prat, Madrid, Spain. The YBZ-1 strain and the pIIIA/MS2-1 plasmid were graciously provided Dr. Marvin Wickens, University of Wisconsin, Madison.

### Screening of RNA-binding proteins

The YBZ-1 yeast strain containing the *StBEL5* full-length 3′ UTR hybrid RNA was transformed with 60 μg of the potato cDNA library. The entire transformation mixture (about 10 mL) was spread onto plates containing SD/-his/-leu/-ura and 1.0 mM 3-aminotrizole (3-AT), a competitive inhibitor of the *HIS3* gene product. To calculate the transformation efficiency, the transformation mixture was serially diluted (10^−1^, 10^−2^, 10^−3^) and grown on small SD/-leu/-ura plates. After 1st and 2nd rounds of screening on different concentration (0, 1, 5, 10, or 50 mM) of 3-AT-containing plates, positive colonies were applied to β-galactosidase assays in order to measure the induction of the *lacZ* reporter gene using a Yeast β-galactosidase Assay Kit (Pierce Biotechnology), according to the manufacturer’s protocol. From the assays, 116 colonies were selected. The 3′ UTR of *StBEL5* with StPTB6-pAD (Mahajan et al., 2012) and an empty pAD were used as positive and negative controls, respectively.

### Plasmid rescue, identification and categorization of the screened clones

In order to identify the positive clones from the screening, yeast plasmid rescue was performed with E.Z.N.A.® Yeast Plasmid Kit (OMEGA bio-tek) with slight modifications. Positive yeast colonies were picked from the plate and inoculated in 3.0 ml of SD/-leu. Overnight grown cells were pelleted and incubated at 30°C for at least 30 min after resuspension in 480 μl Buffer SE/β-mercaptoethanol and 40 μl lyticase solutions. After incubation, yeast plasmid DNA was isolated by following the kit protocols. The rescued yeast plasmids were transformed into *E. coli* HB101 competent cells, and the plasmids were isolated and sequenced using pGAD-specific primers (Table A5 in Appendix) at the DNA Facility, Iowa State University. For putative identities of the clones, sequences of the cDNAs were analyzed at the Dana–Farber Cancer Institute (DFCI) Gene Index^1^ potato database. Translated protein sequences were obtained from Translate^2^ and ORF Finder^3^ and analyzed using BLAST on the TAIR database^4^. Functional categorization of selected proteins was performed using the MIPS *Arabidopsis thaliana* database^5^. The domains of the B5RBPs (Figure 3) were analyzed using SMART^6^ and NCBI’s Conserved Domain Search. For Figure 4A, amino acid sequences of Arabidopsis and potato LSH proteins were organized into a phylogenetic tree with the MEGA 4.0.2 package and the neighbor-joining program. The numbers listed at the branching points are boot-strapping values that indicate the level of significance (percentage) for the separation of two branches.

### RNA gel-shift assays

**Figure A1:**
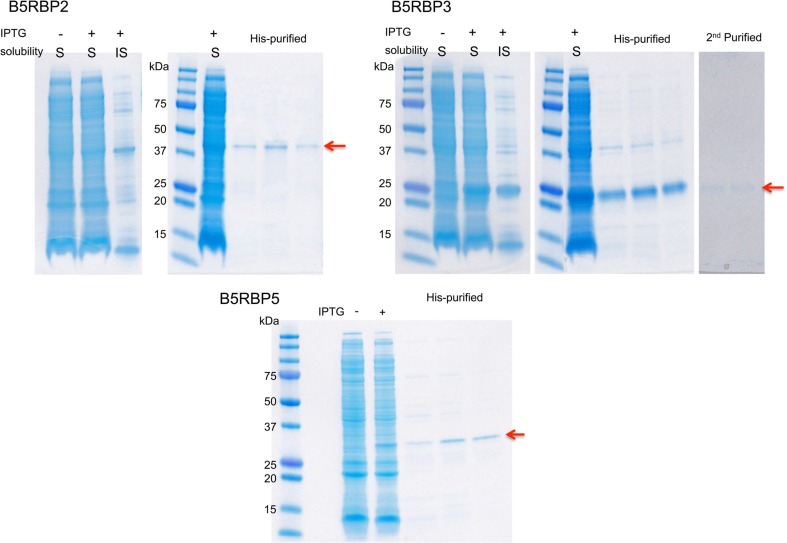
**Coomassie-stained gels of protein purification of three B5RBPs**. Each protein was induced in *E. coli*, the soluble fraction was extracted, and the protein purified once or twice (B5RBP3) using the HisPur Cobalt purification kit (Pierce). The farthest right-hand lanes (arrows) represent from 15 to 100 ng of protein loaded and these samples were used for the RNA EMSAs. (+) indicates the addition of IPTG; (−) no IPTG; S, soluble fraction; IS, insoluble fraction.

The PCR-amplified fragments with gene-specific primer sets (Table A5 in Appendix) were cloned into the pET-28a (+) plasmids after proper enzyme digestion to produce histidine tag (His)-fusion recombinant proteins (Figure A1 in Appendix). The constructs were transformed into *E. coli* BL21-Codon (DE3) cells (Stratagene). The recombinant proteins were induced with 0.4 mM IPTG, and purified using HisPur Cobalt Purification Kit (Pierce Biotechnology). For *in vitro* transcription to generate RNA probes, T3 promoter-containing sense primers were created by adding T3 sequences on the 5′ end of the sense primers of the target sequences (Table A5 in Appendix), and used for PCR amplification using Platinum Taq DNA Polymerase High Fidelity (Invitrogen). The gel-purified PCR product was transcribed using MEGAscript T3 (Ambion) incorporating biotin (biotin-11-UTP, Perkin Elmer) as described by the manufacturer’s manual. The biotin-labeled probe RNA was purified by gel purification using Zymoclean^TM^ Gel RNA Recovery kit (ZymoResearch). Five femtomoles of biotin-labeled RNA probes were incubated with indicated amounts of purified recombinant proteins in the binding buffer provided by the Light Shift Chemiluminescent RNA EMSA kit (Pierce Biotechnology) on ice for 45 min. The RNA-protein complexes were separated in 2.5% agarose (for the full-length probe) or 5% polyacrylamide gel (for the *IRE* probe) and transferred onto BrightStar-Plus (Ambion) nylon membranes. The signal was detected using the EMSA kit according to the manufacturer’s manual.

### Screening for RNA-binding proteins using the yeast three-hybrid system

As eloquently explained by Bernstein et al. (2002), the Y3H system is based on two expression vectors, one for the RNA bait and the other for the protein target, and three-hybrid components. When bait RNA interacts with the target protein, the reporter genes, *HIS3* and *lacZ*, are activated and can be readily detected by simple biochemical assays (Hook et al., 2005; Seay et al., 2006).

**Figure 1:**
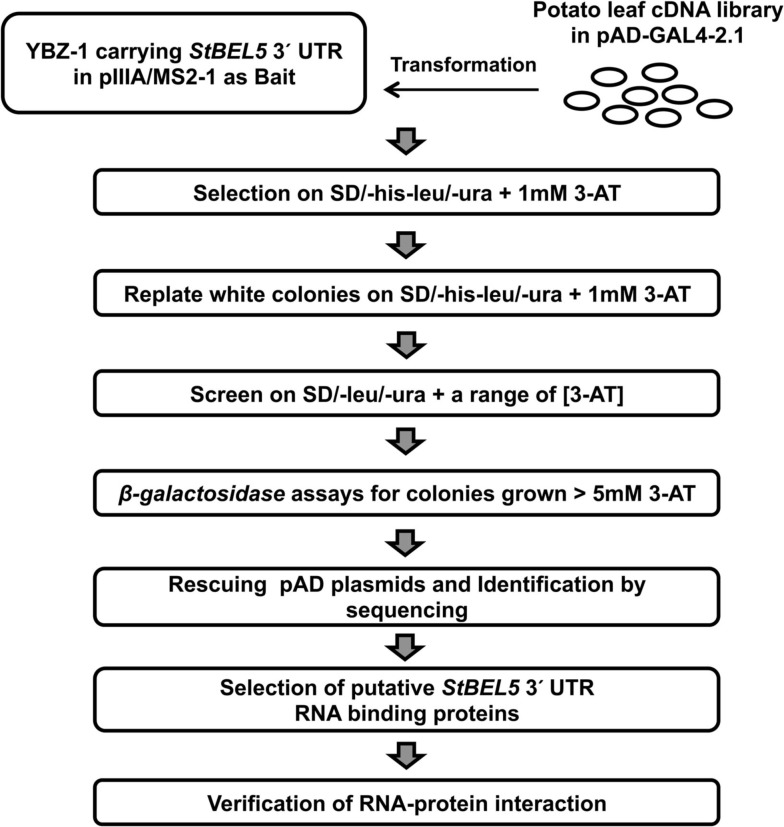
**Schematic diagram of the Y3H system for screening interacting partner proteins from a leaf cDNA library using the 503-nt 3′ UTR of StBEL5 as bait**.

To isolate interacting proteins, the DNA fragment encoding 3′ UTR of *StBEL5* as a bait was cloned into the MS2 portion of pIIIA/MS2-1 vector. The resulting plasmids carrying hybrid *MS2-*3′ UTR of *StBEL5* were transformed into a yeast strain, YBZ-1, and the potato leaf cDNA library was sequentially transformed using conventional protocols with slight modifications (Bernstein et al., 2002; Seay et al., 2006). We analyzed approximately 6.5 × 10^5^ yeast colonies, and the resulting transformed colonies were screened on SD/-his/-leu/-ura plates containing 1.0 mM 3-AT (Figure 1). From the first round, 448 colonies were selected as primary positives. Those selected positive colonies were replicated on SD/-his/-leu/-ura plates containing 1.0 mM 3-AT again to remove potential false positives. From these screenings, 281 colonies were chosen for further screening by using the two reporter genes, *HIS3* and *lacZ*. SD/-his/-leu/-ura plates containing a series of 3-AT concentration (0, 1, 3, 5, 10, and 50 mM) were used for testing *HIS3* expression. The 281 colonies were streaked on these plates, and 194 colonies were grown on SD/-his/-leu/-ura plates containing at least 5.0 mM 3-AT. Finally, 116 colonies were selected based on β-galactosidase and *HIS3* activation and were sequenced and analyzed (Table A1 in Appendix). The overall strategy of the screening is summarized in Figure 1. As a comparison, using the yeast strain YBZ-1, 49 *HIS3*+ colonies were selected out of approximately 8.0 × 10^5^ transformants according to Hook et al. (2005). The interactions of the 3′ UTR of *StBEL5* with StPTB6 and the 3′ UTR of *StBEL5* with empty pGAD vector were used as positive and negative controls for RNA-protein interaction, respectively. PTB proteins are multifunctional proteins that bind numerous mRNAs and are involved in a wide range of RNA metabolism, such as RNA stability, splicing, translational repression, and long-distance transport. There are six PTBs in the potato genome, and one, designated StPTB6, binds to untranslated regions of phloem-mobile mRNAs of potato (Mahajan et al., 2012).

### Characterization of selected cDNA clones

For identification of the screened colonies, their sequences were analyzed and BLAST was performed using the *Arabidopsis* and potato databases, and a total of 89 clones (76.7%) exhibited significant matches to previously characterized or known genes (Table A2 in Appendix). Thirteen clones were identified as redundant (30.2% redundancy), and therefore, a total of 94 unique singletons were isolated from the Y3H screening (listed in Tables A1– A3 in Appendix). These 13 included LSH3 (Light-dependent Short Hypocotyls3), LSH10 (Light-dependent Short Hypocotyls10), C3H zinc finger transcription factor, sucrose synthase4, a Transducin/WD40 repeat-like superfamily protein, LTP12 (Lipid Transfer Protein 12), ELI3-1 (elicitor-activated gene 3-1), X-ray induced transcript 1, glutamate-1-semialdehyde-2, 1-aminomutase, and some ribosomal proteins and unknown proteins (Table A3 in Appendix). Interestingly, LSH10, a close sequence match to AtLSH10 (AT2G42610), was identified from 10 clones along with another LSH member, LSH3, which was isolated twice. Twenty-seven clones (23.3%) were categorized as undefined clones, i.e., “unknown” or “no hit” from the database search (Table A2 in Appendix). Functional classification revealed a total of eight cDNAs that encoded proteins with DNA/RNA-binding properties. Nineteen clones encoded proteins that are components of the machinery for protein synthesis (16.4%) at the initiation and/or elongation of translation, such as eIF5A, transducin/WD40 repeat-like superfamily, heat shock protein 70, and an alpha-tubulin protein (Doroshenk et al., 2009; Lin et al., 2009; Tables A1 and A4 in Appendix). Overall, 35 cDNAs (30.1% of total screened clones) encoding proteins with RNA-binding properties were identified (Table A4 in Appendix).

### Candidate protein partners for *StBEL5* RNA

**Figure 2:**
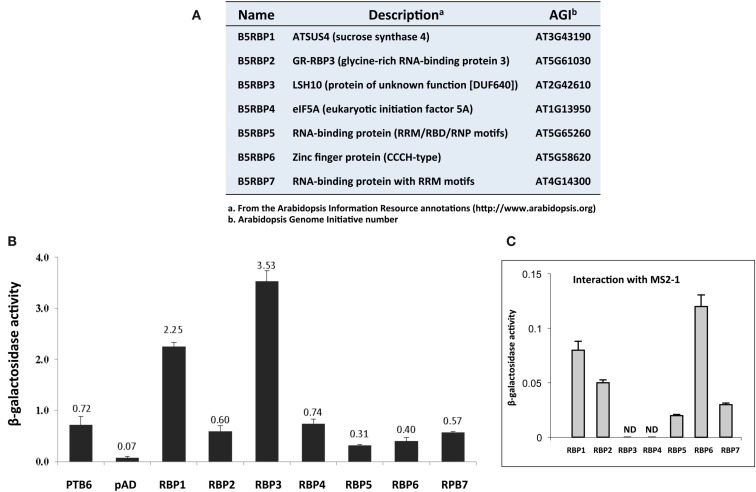
**List of seven B5RBPs (A) and a quantitative analyses of their interactions with the 503-nt 3 ′ UTR of *StBEL5* using a β-galactosidase assay (B)**. The 3′ UTR of *StBEL5* with StPTB6-pAD was used as a positive control **(B)**, and an empty pAD **(B)** or the pIIIA/MS2-1 bait vectors **(C)** were used as negative controls. The numbers above each bar **(B)** represent the means for β*-*galactosidase activity in triplicate, and standard errors are shown for each mean **(B,C)**. The RNA produced by the pIIIA/MS2-1 vector in yeast, including the 60-nt MS2 sites, is approximately 272 nt in length (Bernstein et al., 2002).

**Figure 3:**
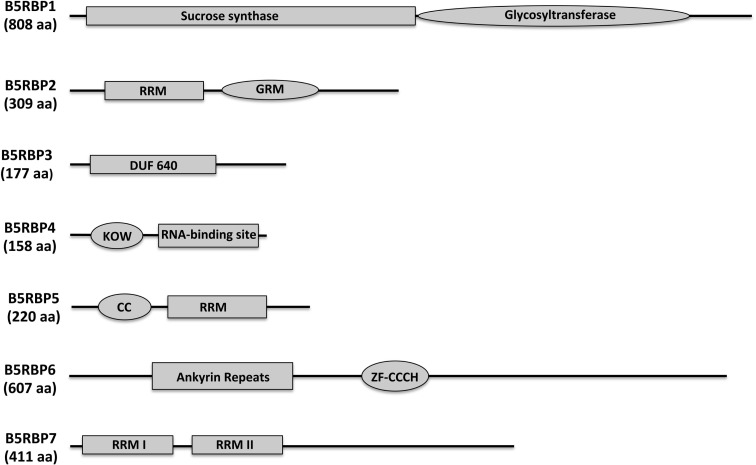
**Protein structure and prediction of conserved domains of the seven B5RBPs**. The deduced amino acid sequences of the B5RBPs were analyzed using SMART (http://smart.embl-heidelberg.de/) and NCBI’s Conserved Domain Search. RRM, RNA recognition motif; DUF, domain of unknown function; GRM, glycine-rich motif; KOW, from Kyrpides et al. (1996); CC, coiled-coil motif; ZF-CCCH, zinc finger, CCCH-type.

Based on activities of marker genes (*HIS3* and *lacZ*), and their putative RNA-binding function, seven cDNA clones were selected for verification as protein partners of *StBEL5*, and designated as *StBEL5* RNA-binding protein (B5RBP) one to seven (Figure 2A). Related proteins of three of the B5RBPs, B5RBP1, -4, and -6, were previously identified as RBPs in other species (Xu et al., 2004; Doroshenk et al., 2009; Ling et al., 2011), and three others, B5RBP2, -5, and -7, contain conserved RNA recognition motifs (RRMs; Figure 3). The most frequently identified clone from the Y3H screening, StLSH10 (B5RBP3), was also included (Table A3 in Appendix). Each of these proteins induced β-galactosidase activity in an interaction with the bait RNA to levels much higher than the negative controls and, in some cases, even higher than the positive control (Figures 2B,C).

*B5RBP1* encodes a sucrose synthase (SUS4), containing a sucrose synthase motif for the sucrose metabolic pathway and a glycosyltransferase motif for biosynthetic processes (Figure 3). There were three reasons to include SUS4. First, it was identified as a cytoskeleton-associated RBP from developing rice seeds (Doroshenk et al., 2009). Second, in *Arabidopsis*, SUS4 was detected in the companion cells of the phloem (Fallahi et al., 2008) and third, it plays an important role in starch metabolism in potato tubers (Fu et al., 1995; Zrenner et al., 1995; Bieniawska et al., 2007). With regard to the role of SUS4 in potato tuber development, high-level increases in *SUS4* promoter activity were observed during early tuber formation (Fu et al., 1995) and transgenic lines overexpressing SUS4 produced enhanced tuber yields (Baroja-Fernandez et al., 2009). SUS4 could be an example of a multifunctional RBP directly involved in potato tuber development.

*B5RBP2* encodes a glycine-rich RNA-binding protein that contains a RRM for RNA-binding and a glycine-rich motif (GRM, Figure 3). Plant proteins that contain a GRM are grouped into five classes based on structure. B5RBP2 is considered a class IV member because it contains a RRM (Mangeon et al., 2010). Class IV GRM-proteins are subdivided based on their links to osmotic stress, cold stress, flower timing, development, and responsiveness to abscisic acid. Interestingly, B5RBP2 is orthologous to AtGRP7 (AT2G21660) a protein related to a RBP found in pumpkin phloem sap (Lin et al., 2009). B5RBP2 may function in potato phloem sap by interacting with *StBEL5* RNA to facilitate long-distance movement. AtGRP7 is also involved in the regulation of alternative splicing, ribosome function, and RNA metabolism (Wachter et al., 2012).

**Figure 4:**
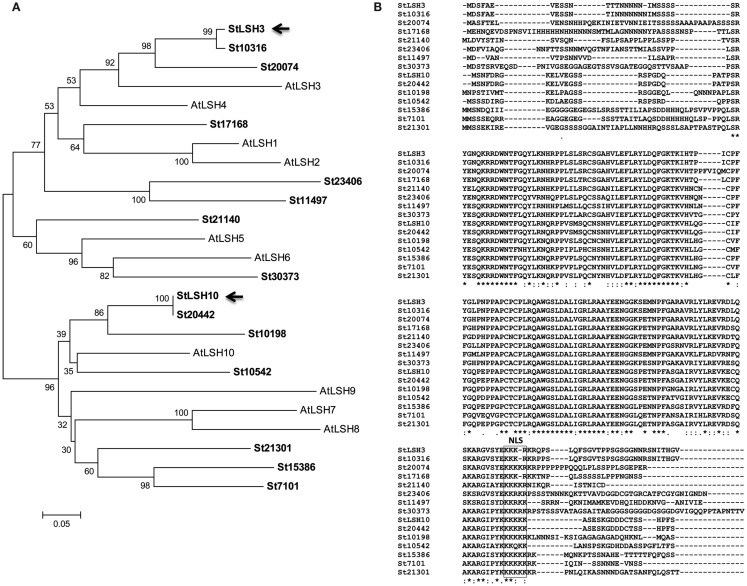
**Phylogenetic analysis (A) and alignment of the aa sequence (B) of the family of LSH proteins of potato (St)**. Included in the dendrogram **(A)** are the 10 LSH proteins of *Arabidopsis* (At). Arrows indicate the two StLSHs, LSH3 and -10, identified from the current yeast three-hybrid screening. The nuclear localization signal (NLS) is boxed **(B)**. Asterisks below the aligned amino acids **(B)** indicate conserved residue identity.

*B5RBP3* is alight-dependent short hypocotyl (LSH10, AT2G42610) protein with a Domain of Unknown Function (DUF640, Figure 3). In *Arabidopsis*, there are 10 LSH genes and in potato, 15 (Figure 4A). The function of most these proteins, ranging in size from 164 to 219 aa, are unknown except for AtLSH1, -3, and -4. AtLSH1 is a nuclear protein in *Arabidopsis* with a nuclear localization signal (NLS) in the C-terminal region. It is involved in light regulation of seedling development (Zhao et al., 2004). Both AtLSH3 and -4 appear to have a role in *Arabidopsis* shoot and floral organ differentiation since constitutive expression of these genes resulted in abnormal development (Takeda et al., 2011). As described earlier, LSH10 was the most frequently selected cDNA from the screen (Table A3 in Appendix). β-galactosidase activity of the B5RBP3/*StBEL5* interaction was several-fold greater than the other B5RBPs (Figure 2B). All of the potato LSH proteins exhibit a highly conserved internal region representative of a domain from the DUF640 superfamily flanked by sequence of considerable variance at both the amino- and carboxy-termini (Figure 4B).

*B5RBP4* encodes a eukaryotic initiation factor 5A (eIF5A, AT1G13950) containing both KOW (acronym of the authors surname, Kyrpides et al., 1996; Figure 3) and eIF5A motifs for ribosome binding, RNA-binding, and translation activity (Figure 3). Studies in bacteria suggest that the KOW motif mediates RNA and protein interactions (Steiner et al., 2002). For simplicity, the eIF5A motif is referred to as a RNA-binding site (Figure 3) since the motif is characterized as a S1-like RNA-binding domain (Peat et al., 1998). eIF5A is a multifunctional protein involved in RNA-binding, processing, turnover, and transport from the nucleus to cytoplasm and in transcription and translation (Burd and Dreyfuss, 1994; Cusack, 1999; Xu and Chen, 2001). Recently, eIF5A in yeast was shown to stimulate protein synthesis but was not required for the process (Henderson and Hershey, 2011). In pumpkin phloem sap, CmeIF5A was detected as a component of the RBP50-based ribonucleoprotein complex (Ham et al., 2009). Further characterization of CmelF5A revealed that hypusination (lysine residue modification) was necessary for RNA-binding and protein interaction and that both hypusinated and non-hypusinated CmelF5A existed in the phloem (Ma et al., 2010). B5RBP4 may function through a similar mechanism in potato phloem sap to mediate the formation of ribonucleoprotein complexes.

*B5RBP5* encodes a RNA-binding (RRM/RNA-Binding Domain/Ribonucleoprotein → RRM/RBD/RNP) protein family member containing a coiled-coil motif (CC) and a RRM (Figure 3) that is orthologous to At5G65260, an *Arabidopsis* RNA-binding protein. At5G65260 is designated as a polyadenylation factor that can bind to the poly (A) tail and control its length (Hunt et al., 2008). The *Arabidopsis* transcription factor Long Hypocotyl5 (HY5) that is involved in photomorphogenesis was shown to mediate the expression of At5G65260 (Lee et al., 2007). At5G65260 expression was down-regulated in a loss-of-function HY5 mutant. It is conceivable that B5RBP5 levels in potato may also be regulated by light.

*B5RBP6* encodes a zinc finger (CCCH-type) family protein containing two ankyrin repeats for protein-protein interactions, and two zinc finger-C3H1 domains (ZF-CCCH) for zinc ion binding, and nucleic acid binding (Figure 3). Unlike other zinc finger proteins that generally function as DNA-binding proteins (Laity et al., 2001), CCCH zinc finger proteins bind to AU-rich elements of RNAs (Brown, 2005). A study in mouse revealed a role for a CCCH zinc finger protein (tristetraprolin) in mRNA decay (Lai et al., 2006). In *Trypanosoma brucei*, the causative agent of sleeping sickness, the CCCH zinc finger protein (Tb2C3H20) functions in mRNA stability (Ling et al., 2011). In *Arabidopsis*, two CCCH-type zinc finger genes, designated AtSZF1, and AtSZF2, were salt-inducible and mediated responsiveness to salt (Sun et al., 2007). From the current screen, two CCCH-type zinc finger proteins were identified (Table A4 in Appendix) that shared sequence similarity with AT2G40140 (AtSZF2) and AT5G58620 from *Arabidopsis*.

*B5RBP7* encodes another RNA-binding (RRM/RBD/RNP motifs) family protein that shares sequence similarity with *Arabidopsis* AtRNP1 (AT4G14300) containing two RRM domains (Figure 3). AtRNP1 is a target of *Arabidopsis* transportin 1 (AtTRN1) that is an ortholog of the human nuclear import receptor transportin1 protein (Ziemienowicz et al., 2003). AtRNP1 may function as a shuttle protein moving RNAs between the nucleus and cytoplasm. Interestingly, AtGRP7 also interacted with AtTRN1. As discussed above, B5RBP2 is orthologous to AtGRP7 suggesting that B5RBP7 and B5RBP2 may associate with *StBEL5* RNA as a tandem complex.

### Expression profiles

To assess transcript levels for select RBPs, expression values were obtained from the publicly available RNA-seq database from the RH genotype of the Potato Genome Sequencing Project (Xu et al., 2011). Abundance levels of *StBEL5* and *StHSP70* have been included as references. The potato HSP70 protein was selected during the screen (Table A4 in Appendix) and a HSP70-type was previously identified as a member of a phloem-mobile RNP complex in pumpkin (Ham et al., 2009). Relatively high and consistent levels of transcripts across all organs were observed for B5RBP2, -4, HSP70, and both RBPs, B5RBP5, and -7 (Table 1). The very high levels of eIF5A (B5RBP4) likely reflect its general, multifunctional role in several aspects of RNA metabolism (Zanelli and Valentini, 2007). B5RBP1 (sucrose synthase) scored abundant RNA levels in both stolons and young tubers indicative of its role in starch metabolism during tuber formation. The zinc finger CCCH protein (B5RBP6) was most abundant in petioles with a value of 334 FPKMs (fragments per kb per million mapped reads). A mobile RNA like *StBEL5* is very abundant in petioles, an observation that is consistent with both its transcriptional source and its capacity to move long-distances through the phloem (Banerjee et al., 2006). Petioles serve two main functions: to provide support for the leaf lamina and to act as a protective sleeve for phloem cells that move sugar and signaling molecules (like RNA) from source leaves to sinks. RBPs are commonly detected in companion cells and sieve elements of leaf veins in position to chaperone mobile RNAs (Ham et al., 2009).

**Table 1:** **Expression profile of select B5RBPs mined using the RNA-seq data from the publically available Potato Genome Database (Xu et al., 2011)**.

Gene	Flower	*In vitro* plant	Sprout	Leaf	Petiole	SAM	Stem	Stolon	Young tuber	Root
B5RBP1	43	170	120	10	39	46	62	175	407	151
B5RBP2	91	144	191	129	152	201	113	252	257	165
B5RBP3	0	14	33	0	77	23	46	149	590	53
StLSH3	4	15	12	0	27	5	3	17	3	11
B5RBP4	375	364	414	354	511	199	377	420	418	606
B5RBP5	76	68	93	40	83	63	70	65	71	92
B5RBP6	67	91	46	103	334	48	59	70	40	104
B5RBP7	119	229	392	111	250	188	141	184	184	238
StHSP70	65	64	108	44	100	122	140	236	192	160
StBEL5	35	52	60	39	170	25	77	24	55	42

*Nine organs and an *in vitro* plantlet are presented and abundance values are shown in FPKMs (fragments per kb per million mapped reads). *StBEL5* is shown as a relative control. The potato HSP70 protein is included here because it was selected during the screen (Table A4 in Appendix) and previously was identified as a member of a phloem-mobile RNP complex in pumpkin (Ham et al., 2009)*.

Despite the observation that B5RBP3 (LSH10) appeared 10 times from the Y3H screening (Table A3 in Appendix), its RNA levels were remarkably low in leaf RNA of the RH genotype (Table 1). Similar results for LSH10 RNA abundance levels were observed in RNA-seq data from the DM genotype (Xu et al., 2011). Among the proteins selected, this putative RBP exhibited the strongest induction of β-galactosidase activity (Figure 2). Whereas the second LSH protein selected in this screen, StLSH3, exhibited very low transcript values across all organs, transcript abundance values for B5RBP3 (Table 1) were extremely high in petioles (77 FPKMs), stolons (149 FPKMs), and young tubers (590 FPKMs). This latter transcript value was the second most abundant of any of the RNAs scored in this experiment.

### Verification of RNA-protein interaction of the B5RBPs

**Figure 5:**
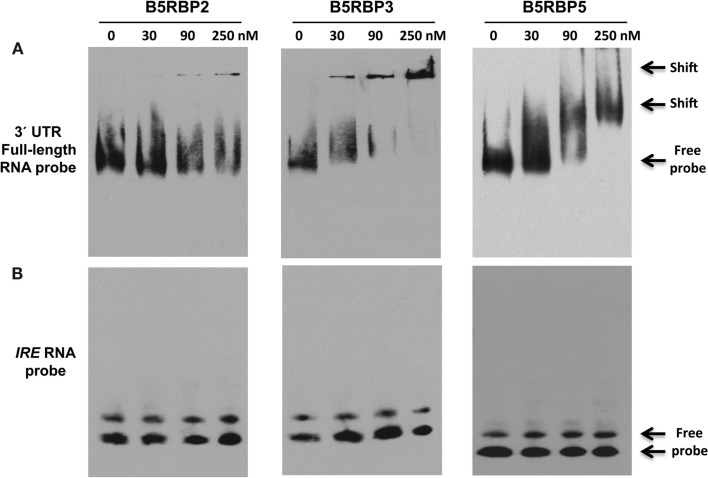
**Mobility shift assays for in the vitro interaction of the 3′ UTR of StBEL5 with select B5RBPs**. **(A)** Full-length 3′ UTR of *StBEL5* with B5RBP2, -3, and -5. The *iron response element* (*IRE)* is included as a negative control **(B)**. Approximately 5 fmole of biotin-labeled bait RNA and protein concentrations ranging from 30 to 250 nM were used in each reaction.

To validate the direct interaction of select proteins with the 3′ UTR of *StBEL5*, RNA gel-shift assays were performed with select B5RBPs. These assays were performed using biotin-incorporated RNA probes using the full-length 3′ UTR of *StBEL5* and the purified recombinant B5RBP2, -3, and -5 proteins (Figure 5A). For showing specificity of the interaction, *IRE* RNA, which binds specifically with the iron responsive protein in the cell under iron-starved conditions, was used as a negative control (Figure 5B). Shifted bands were observed for all three interactions in a range of 30–250 nM of protein. B5RBP3 affected a shift with protein amounts as low as 30 nM, whereas shifted bands were clearly observed with the other two B5RBPs in the reactions containing 90 nM of protein. With comparable amounts of protein, no gel-shift was observed for the negative control, the iron responsive element (*IRE*, Figure 5B).

**Figure 6:**
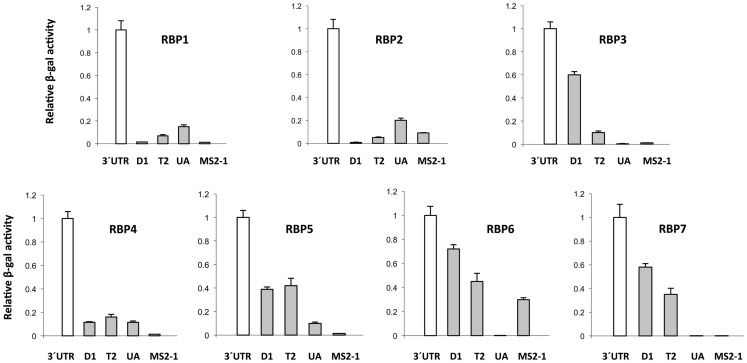
**Analysis of β-galactosidase activity for the interaction of select *StBEL5* RNA-binding proteins (B5RBPs) with sequences within the 3′ UTR of *StBEL5***. The values in each graph are normalized to activity relative to the full-length UTR. 3′ UTR, full-length UTR; D1; a 178-nt sequence within the UTR starting from the stop codon; T2, a UAGU-rich region within the UTR; UA, UA-rich region toward the 3′ end of the UTR; MS2-1, RNA sequence from the bait vector serving as a negative control. See Figure 7 for details on these truncated *StBEL5* bait sequences.

**Figure 7:**
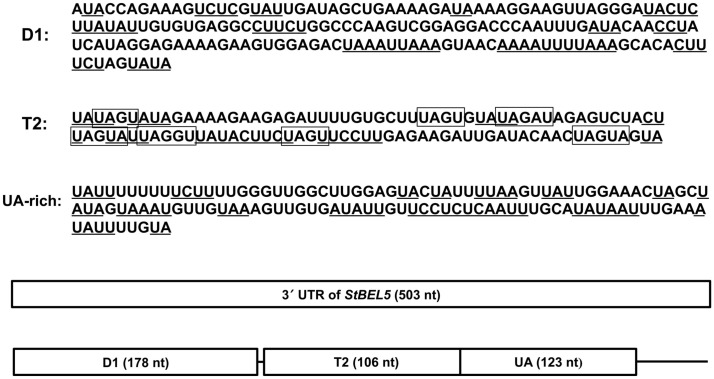
**Schematic diagram of the bait RNA sequences from the 3′ UTR of *StBEL5* used in Figure 6**. Included are three truncated sequences within the full-length 503-nt 3′ UTR: the 178-nt D1 sequence, the 106-nt T2 region, and the 123-nt UA-rich region (UA). Both UA-and CU-rich motifs are underlined. The UAGU motifs of the T2 sequence are boxed.

The 3′ UTR of *StBEL5* is involved in several aspects of RNA metabolism and is replete with potential binding motifs (Banerjee et al., 2009). To identify shorter binding regions within the 3′ UTR that may be involved in protein/RNA interaction, truncated bait sequences were utilized in the β*-*galactosidase assay of the Y3H system (Figure 6). Three truncated sequences were used based on their conserved sequence motifs and their coverage of the UTR (Figure 7). The 5′ D1 sequence is enriched for CU motifs (underlined sequence, Figure 7). T2 contains several UAGU motifs (Figure 7, boxed), and the UA-bait sequence contains a number of uracil/adenine runs (underlined sequence, Figure 7). Overall, the greatest β*-g*alactosidase activity was observed for the full-length 3′ UTR (Figure 6). Based on β*-g*alactosidase activity, B5RBP3, -5, -6, and -7 exhibited the strongest interaction with sequence located toward the 5′ end of the UTR (D1 and T2 baits). B5RBP1 and -2 exhibited the strongest interaction with sequence located toward the 3′ end of the UTR (T2 and UA baits). B5RBP4, the potato ortholog of eIF5A, exhibited equivalent strength of activity with all three truncated baits suggesting a degree of non-specific binding. These results with the potato eIF5A are consistent with previous work showing that a pumpkin form of eIF5A exhibited RNA-binding that was non-sequence-specific in nature (Ma et al., 2010).

## Conclusion

The Y3H system has been established as an efficient method for selecting protein partners of RNA from among thousands of putative partners, and for assaying binding affinity of specific RNA/protein interactions. With modifications, this system has been adapted for screening RNA/RNA interactions (Piganeau and Schroeder, 2006), for identifying protein/small signaling molecule complexes (Cottier et al., 2011), and for testing multi-component interactions (Bernstein et al., 2002). Although numerous false positives may arise during the screening process, there are several levels of selection that may be utilized to eliminate these. These include nutrient selection, *HIS3* activation, addition of 3-aminotriazole, and 5-fluororotic acid to the media, and the induction of *lacZ*. As shown previously (Hook et al., 2005), both *HIS3* and *lacZ* expression levels are directly related to binding affinity and may be used to assess the robustness of specific RNA/protein interactions (Mahajan et al., 2012) or to map specific motifs present in either bait or target sequences (Edwards et al., 2001; Mori et al., 2008; Stumpf et al., 2008).

Factors known to affect *in vivo* interactions include the intrinsic affinity of bait RNA and target protein, the length of the bait RNA, and accessibility of the insert to the target (Wurster and Maher III, 2010). RNA sequences less than 150 nt generally produce the most substantial and specific reporter activation. The inclusion of additional sequence can lead to a reduction in signal. In this study, the entire 503-nt 3′ UTR of *StBEL5* was utilized to include a wide range of interactions. With this approach, subsequent analyses of shorter RNA sequences in RNA/protein interactions using either RNA gel-shift or Y3H assays may be used to identify the location of specific binding motifs. The source of the protein expression library will also play an important role in identification of protein partners. Here the use of a leaf cDNA library would provide wide coverage but could preclude the identification of important interactions with rare phloem-mobile proteins. Three of the RBPs compiled in the final list here (B5RBP1, -5, and -7) appear to function in the intracellular transport of RNAs. Two RBPs identified in the current study, eIF5A and HSP70, were isolated from a phloem-mobile RNP complex of pumpkin (Ham et al., 2009). A protein very close in sequence match to the glycine-rich RBP (B5RBP2) was also identified in pumpkin phloem sap (Lin et al., 2009). Both binding affinity and abundance of the target protein would impact the results of the Y3H screen. As demonstrated by StLSH10, despite its relatively low transcript level in leaves (Table 1), its affinity for the *BEL5* UTR bait was demonstrated to be relatively high. The very high transcript levels observed for StLSH10 (B5RBP3) in stolons and young tubers (149 and 590 FPKMs, respectively) also suggest a possible role in tuber development. Screening a library of proteins from a sink organ like the tuberizing stolon would further expand our understanding of the RNP complex responsible for delivery of the mobile *StBEL5* transcript to its functional site. Overall, these results demonstrate the utility of the Y3H system in screening and verifying interactions between target RNAs and putative RBPs.

## Conflict of Interest Statement

The authors declare that the research was conducted in the absence of any commercial or financial relationships that could be construed as a potential conflict of interest.

## Footnotes

^1^http://compbio.dfci.harvard.edu/cgibin/tgi/gimain.pl?gudb = potato

^2^http://web.expasy.org/translate/

^3^www.ncbi.nlm.nih.gov/gorf/gorf.html

^4^http://www.arabidopsis.org

^5^http://mips.helmholtz-muenchen.de

^6^http://smart.embl-heidelberg.de/

## Appendix

**Table A1:** **Full list of the screened genes with their putative identities and concentration of 3-AT for screening**.

Clone no	AGI^a^	Description^b^	*E*-value	Potato^c^	3-AT^d^
#1–1	AT4G16260	Glycosyl hydrolase superfamily protein	2E−96	TC194747	50
#1–9	AT4G17880	Basic helix-loop-helix (bHLH) DNA-binding family protein	3E−66	TC207090	50
#1–12	AT2G37190	Ribosomal protein L11	3E−71	TC200521	10
#1–21	AT3G43190	ATSUS4 | sucrose synthase 4	0	TC194622	50
#1–22	AT3G43190	ATSUS4 | sucrose synthase 4	0	TC194622	10
#1–25	AT2G38040	Acetyl co-enzyme a carboxylase carboxyltransferase alpha subunit	2E−76	TC202772	5
#1–38	AT2G40140	ATSZF2 (salt-inducible zinc finger 2)	8E−23	TC208753	10
#1–40	AT4G05320	Polyubiquitin 10	0	TC204873	5
#1–55	AT1G58684	Ribosomal protein S5	E−111	TC225313	5
#1–57	AT4G15470	Bax inhibitor-1	1E−71	TC200584	5
#1–61	AT3G14610	Cytochrome P450, family 72, subfamily A, polypeptide 7	E−124	TC197461	5
#2–5	AT3G12120	Fatty acid desaturase 2	E−143	TC197103	50
#2–23	AT5G57280	*S*-Adenosyl-l-methionine-dependent methyltransferases	9E−77	AW906478	50
#2–35	AT1G26830	ATCUL3A (cullin 3)	0	TC220451	5
#2–36	AT5G24530	2-Oxoglutarate (2OG) and Fe(II)-dependent oxygenase	8E−37	TC212020	50
#2–51	AT2G23940	Protein of unknown function (DUF788)	6E−60	TC223843	5
#2–52	AT5G48720	X-ray induced transcript 1	1E−26	BG597043	50
#2–57	AT1G47260	APFI/gamma carbonic anhydrase 2	E−126	TC195322	5
#2–62	AT5G48720	XRI, XRI1 | X-ray induced transcript 1	6E−17	BG597043	5
#3–1	AT3G18000	*S*-Adenosyl-l-methionine-dependent methyltransferases	0	TC212421	10
#3–4	AT3G13340	Transducin/WD40 repeat-like superfamily protein	3E−92	TC222486	10
#3–7	AT1G58684	Ribosomal protein S5	E−111	TC225313	5
#3–16	AT1G53840	ATPME1, PME1 | pectin methylesterase 1	9E−20	EG012025	5
#3–20	AT3G05590	Ribosomal protein L18	8E−83	TC210667	5
#3–33	AT4G37980	ELI3-1, ELI3, ATCAD7, CAD7 | elicitor-activated gene 3-1	4E−20	TC223852	5
#3–39	AT5G61030	Glycine-rich RNA-binding protein	2E−30	TC203116	50
#3–43	AT2G46500	ATPI4K GAMMA 4 (phosphoinositide 4-kinase gamma 4)	3E−54	TC208976	5
#3–53	AT1G49040	SCD1 | stomatal cytokinesis defective/SCD1 protein: WD40 containing	2E−63	TC206273	50
#4–1	AT5G19510	Translation elongation factor EF1B/ribosomal protein S6 family protein	2E−26	TC197463	5
#4–5	AT2G33220	GRIM-19 protein	1E−63	TC201317	5
#4–13	AT4G22140	EBS | PHD finger family protein/bromo-adjacent homology (BAH) domain-containing	6E−98	TC216086	5
#4–33	AT5G52640	Heat shock protein 90	E−157	TC200319	10
#4–36	AT1G22410	Class-II DAHP synthetase family protein	0	TC194919	5
#4–37	AT5G39510	Vesicle transport v-SNARE family protein	6E−08	TC196409	5
#4–60	AT3G43120	SAUR-like auxin-responsive protein family	7E−19	TC219427	5
#5–1	AT2G42210	ATOEP16-3, OEP16-3 | Mitochondrial import inner membrane translocase subunit	2E−54	TC204735	50
#5–2	AT3G01320	SNL1 | SIN3-like 1	1E−42	CV286681	10
#5–4	AT5G27700	Ribosomal protein S21e	4E−43	TC214159	5
#5–5	AT5G25610	RD22, ATRD22 | BURP domain-containing protein	1E−32	TC201553	10
#5–6	AT1G03860	ATPHB2, PHB2 | prohibitin 2	E−120	TC196152	50
#5–9	AT4G09800	S18 ribosomal protein	5E−44	TC207656	10
#5–10	AT2G42610	LSH10 | Protein of unknown function (DUF640)	1E−64	TC202398	10
#5–11	AT2G42610	LSH10 | Protein of unknown function (DUF640)	1E−64	TC202398	10
#5–12	AT1G49310	Unknown protein	0.002	TC207198	5
#5–13	AT3G60520	Unknown protein	0.047	TC196277	50
#5–17	AT1G27450	APT1, ATAPT1 | adenine phosphoribosyl transferase 1	2E−78	TC196278	5
#5–21	AT3G50500	SNRK2.2 | SNF1-related protein kinase 2.2	3E−15	EG012856	5
#5–22	AT1G60420	DC1 domain-containing protein	E−100	TC199573	50
#5–23	AT1G67785	Unknown protein	2E−16	TC216692	5
#5–24	AT1G22100	Inositol-pentakisphosphate 2-kinase family protein	1E−67	TC213605	5
#5–26	AT5G26220	ChaC-like family protein	1E−79	TC195543	10
#5–27	AT2G42610	LSH10 | Protein of unknown function (DUF640)	2E−60	TC205325	10
#5–29	AT2G42610	LSH10 | Protein of unknown function (DUF640)	5E−14	TC205325	50
#5–30	AT1G19580	GAMMA CA1 | gamma carbonic anhydrase 1	E−129	TC196517	5
#5–31	AT1G76670	Nucleotide-sugar transporter family protein	E−147	TC198970	10
#5–32	AT1G13950	EIF-5A, ELF5A-1, ATELF5A-1, EIF5A | eukaryotic elongation factor 5A-1	3E−72	TC202667	5
#5–34	AT2G42610	LSH10 | Protein of unknown function (DUF640)	2E−60	TC202398	10
#5–35	AT2G23610	ATMES3, MES3 | methyl esterase 3	5E−41	TC221190	10
#5–37	AT3G25660	Amidase family protein	2E−73	CV506455	50
#5–38	AT1G18080	ATARCA (Transducin/WD40 repeat-like superfamily protein)	E−118	TC197724	5
#5–39	AT2G31160	LSH3 | Protein of unknown function (DUF640)	1E−54	CV502385	50
#5–42	AT2G42610	LSH10 | Protein of unknown function (DUF640)	1E−64	TC202398	50
#5–43	AT4G28940	Phosphorylase superfamily protein	E−113	TC195965	5
#5–44	AT2G42610	LSH10 | Protein of unknown function (DUF640)	2E−70	TC202398	10
#5–46	AT5G65020	ANNAT2 | annexin 2	E−88	TC208485	5
#5–48	AT5G02380	MT2B | metallothionein 2B	0.007	TC217979	50
#6–1	AT3G15610	Transducin/WD40 repeat-like superfamily protein	3E−34	TC199143	5
#6–2	AT1G25520	Uncharacterized protein family (UPF0016)	3E−88	TC215875	5
#6–4	AT2G19730	Ribosomal L28e protein family	4E−59	TC200167	5
#6–5	AT3G16240	DELTA-TIP, TIP2;1, DELTA-TIP1, AQP1, ATTIP2;1	3E−77	TC195833	5
#6–6	AT2G42610	LSH10 | Protein of unknown function (DUF640)	1E−64	TC202398	10
#6–9	AT2G31160	LSH3 | Protein of unknown function (DUF640)	2E−54	DR034011	5
#6–10	AT1G58684	Ribosomal protein S5 family protein	E−111	TC225313	5
#6–14	AT2G42610	LSH10 | Protein of unknown function (DUF640)	1E−64	TC202398	10
#6–19	AT1G36320	Unknown protein	2E−72	TC198511	5
#6–20	AT5G59240	Ribosomal protein S8e family protein	4E−80	TC205286	5
#6–24	AT5G63570	GSA1 | glutamate-1-semialdehyde-2,1-aminomutase	E−115	TC210671	5
#6–25	AT2G42610	LSH10 | Protein of unknown function (DUF640)	8E−65	TC196879	10
#6–26	AT1G63000	NRS/ER, UER1 | nucleotide-rhamnose synthase/epimerase-reductase	E−151	TC194991	5
#6–30	AT1G43170	RP1 | ribosomal protein 1	0	TC196190	5
#6–31	AT5G65260	RNA-binding (RRM/RBD/RNP motifs) family protein	E−60	TC196348	5
#6–35	AT3G04400	Ribosomal protein L14p/L23e family protein	4E−69	TC208592	5
#6–37	AT4G14960	TUA6 | Alpha-tubulin/FtsZ family protein	0	TC196128	5
#6–38	AT5G54770	THI1, TZ, THI4 | thiazole biosynthetic enzyme, chloroplast (ARA6; THI1; THI4)	E−114	TC194928	5
#6–42	AT5G01870	Bifunctional inhibitor/lipid transfer protein/seed storage 2S albumin superfamily protein	4E−13	TC213303	5
#6–44	AT1G27400	Ribosomal protein L22p/L17e family protein	8E−70	TC214142	5
#7–1	AT3G15610	Transducin/WD40 repeat-like superfamily protein	E−120	TC204693	5
#7–2	AT1G54290	Translation initiation factor SUI1 family protein	6E−51	TC202798	5
#7–3	AT5G62220	ATGT18, GT18 | glycosyltransferase 18	E−131	TC195956	5
#7–7	AT1G79920	Heat shock protein 70 (Hsp 70) family protein	E−133	TC204446	5
#7–8	AT1G18540	Ribosomal protein L6 family protein	3E−75	TC198860	5
#7–9	AT1G18540	Ribosomal protein L6 family protein	2E−67	TC198860	5
#7–14		No Hit		CK861625	5
#7–15	AT3G06030	ANP3, MAPKKK12, NP3 | NPK1-related protein kinase 3	E−39	CK863139	5
#7–18	AT1G26355	SP1L1 | SPIRAL1-like1	9E−22	TC208981	5
#7–19	AT5G64300	ATGCH, GCH, ATRIBA1, RFD1 | GTP cyclohydrolase II	0	TC207697	5
#7–22	AT5G58620	Zinc finger (CCCH-type) family protein	E−144	TC196771	5
#7–23	AT5G63570	Glutamate-1-semialdehyde 2,1-aminomutase	E−115	TC210671	5
#7–28	AT4G23895	Pleckstrin homology (PH) domain-containing protein	2E−97	TC194970	5
#7–29	AT1G11680	Putative obtusifoliol 14-alpha demethylase involved in sterol biosynthesis	E−169	TC202830	5
#7–32	AT2G47730	Glutathione transferase belonging to the phi class of GSTs	4E−69	TC218491	5
#7–33	AT3G25660	Amidase family protein	2E−73	CV506455	5
#7–35	AT4G13350	NIG | NSP (nuclear shuttle protein)-interacting GTPase	4E−07	TC200976	5
#7–36	AT1G07890	Cytosolic ascorbate peroxidase APX1	E−116	TC195529	5
#7–37	AT4G14300	RNA-binding (RRM/RBD/RNP motifs) family protein; AtRNP1	9.00E−73	TC200460	5
#7–40	AT1G14685	BPC2, BBR/BPC2, ATBPC2 | basic pentacysteine 2	3E−62	TC202087	5
#7–41	AT4G38510	ATPase, V1 complex, subunit B protein	0	TC197270	5
#7–42	AT4G37980	Elicitor-activated gene 3-1 (ELI3-1)	5E−20	TC223852	5
#7–46	AT1G36320	Unknown protein	2E−76	TC198511	5
#8–4	AT3G51590	Lipid transfer protein 12 (LTP12)	2E−14	TC221371	5
#8–17	AT4G29410	Ribosomal L28e protein family	1E−46	TC200167	5
#8–26	AT3G53120	VPS37-1 | Modifier of rudimentary (Mod(r)) protein	2E−47	TC205847	5
#8–30	AT4G00100	ATRPS13A, RPS13, PFL2, RPS13A | ribosomal protein S13A	2E−78	TC197315	5
#8–44	AT5G49650	Xylulose kinase 2	E−132	TC211252	5
#8–45	AT3G51590	Lipid transfer protein 12 (LTP12)	2E−14	TC221371	5
#8–47	AT1G67325	Ran BP2/NZF zinc finger-like superfamily protein	8E−98	TC194820	5

*^a^Arabidopsis Genome Initiative number*.

*^b^From the *Arabidopsis* Information Resource annotations (http://www.arabidopsis.org)*.

*^c^From DFCI Database*.

*^d^Concentration of 3-AT (mM) for screening the clones*.

**Table A2:** **Summary of clones from the yeast three-hybrid screening**.

	Number	Ratio (%)
**Total screened clones**	116	
BLAST-match clones	89	76.7
BLAST-no match clones	27	23.3
Non-redundant clones	81	69.8
Redundant clones	35	30.2
Total singletons	94	
**Functions**
DNA/RNA-binding	8	6.9
Protein synthesis	19	16.4
Metabolism	28	24.1
Transcription activity	7	6.0
Structures	18	15.5
Others	14	12.1
Unknown/uncharacterized	27	23.3

*The number of the clones and their percentages are presented*.

**Table A3:** **List of the redundant clones and their identities**.

AGI^a^	Description^b^	Potato^c^	No of clones
AT2G42610	LSH10 | Protein of unknown function (DUF640)	TC202398	10
AT1G58684	Ribosomal protein S5	TC225313	3
AT1G18540	Ribosomal protein L6 family protein	TC198860	2
AT1G36320	Unknown protein	TC198511	2
AT2G31160	LSH3 | Protein of unknown function (DUF640)	CV502385	2
AT2G40140	ATSZF2 (salt-inducible zinc finger 2)	TC208753	2
AT3G15610	Transducin/WD40 repeat-like superfamily protein	TC199143	2
AT3G25660	Amidase family protein	CV506455	2
AT3G43190	ATSUS4 | sucrose synthase 4	TC194622	2
AT3G51590	Lipid transfer protein 12 (LTP12)	TC221371	2
AT4G37980	ELI3-1, ELI3, ATCAD7, CAD7 | elicitor-activated gene 3-1	TC223852	2
AT5G48720	X-ray induced transcript 1	BG597043	2
AT5G63570	Glutamate-1-semialdehyde 2,1-aminomutase	TC210671	2

*^a^Arabidopsis Genome Initiative number*.

*^b^From the Arabidopsis Information Resource annotations (http://www.arabidopsis.org)*.

*^c^From DFCI Database*.

**Table A4:** **List of the screened clones with RNA-binding properties**.

Clone No	AGI^a^	Description^b^	Property
#1–9	AT4G17880	Basic helix-loop-helix (bHLH) DNA-binding family protein	DNA/RNA-binding
#1–38	AT2G40140	ATSZF2 (salt-inducible zinc finger 2)	DNA/RNA-binding
#3–39	AT5G61030	StGRP3 | glycine-rich RNA-binding protein 3	DNA/RNA-binding
#4–13	AT4G22140	EBS | PHD finger family protein	DNA/RNA-binding
#6–31	AT5G65260	RNA-binding (RRM/RBD/RNP motifs) family protein	DNA/RNA-binding
#7–22	AT5G58620	Zinc finger (CCCH-type) family protein	DNA/RNA-binding
#7–37	AT4G14300	RNA-binding (RRM/RBD/RNP motifs) family protein; AtRNP1	DNA/RNA-binding
#7–40	AT1G14685	BPC2, BBR/BPC2, ATBPC2 | basic pentacysteine 2	DNA/RNA-binding
#1–21	AT3G43190	ATSUS4 | sucrose synthase 4	RBP
#1–22	AT3G43190	ATSUS4 | sucrose synthase 4	RBP
#3–4	AT3G13340	Transducin/WD40 repeat-like superfamily protein	RBP
#5–38	AT1G18080	ATARCA (Transducin/WD40 repeat-like superfamily protein)	RBP
#6–1	AT3G15610	Transducin/WD40 repeat-like superfamily protein	RBP
#6–37	AT4G14960	TUA6 | Alpha-tubulin/FtsZ family protein	RBP
#7–1	AT3G15610	Transducin/WD40 repeat-like superfamily protein	RBP
#7–7	AT1G79920	Heat shock protein 70 (Hsp 70) family protein	RBP
#1–12	AT2G37190	Ribosomal protein L11	Translation
#1–55	AT1G58684	Ribosomal protein S5	Translation
#3–7	AT1G58684	Ribosomal protein S5	Translation
#3–20	AT3G05590	Ribosomal protein L18	Translation
#4–1	AT5G19510	Translation elongation factor EF1B/ribosomal protein S6 family protein	Translation
#5–4	AT5G27700	Ribosomal protein S21e	Translation
#5–9	AT4G09800	S18 ribosomal protein	Translation
#6–4	AT2G19730	Ribosomal L28e protein family	Translation
#6–10	AT1G58684	Ribosomal protein S5 family protein	Translation
#6–20	AT5G59240	Ribosomal protein S8e family protein	Translation
#6–30	AT1G43170	RP1 | ribosomal protein 1	Translation
#6–35	AT3G04400	Ribosomal protein L14p/L23e family protein	Translation
#6–44	AT1G27400	Ribosomal protein L22p/L17e family protein	Translation
#7–2	AT1G54290	Translation initiation factor SUI1 family protein	Translation
#7–8	AT1G18540	Ribosomal protein L6 family protein	Translation
#7–9	AT1G18540	Ribosomal protein L6 family protein	Translation
#8–17	AT4G29410	Ribosomal L28e protein family	Translation
#8–30	AT4G00100	ATRPS13A, RPS13, PFL2, RPS13A | ribosomal protein S13A	Translation
#5–32	AT1G13950	EIF-5A, ELF5A-1, ATELF5A-1, EIF5A | eukaryotic elongation factor 5A-1	Translation/RBP

*^a^Arabidopsis Genome Initiative number*.

*^b^From the Arabidopsis Information Resource annotations (http://www.arabidopsis.org)*.

**Table A5:** **List of primers**.

Name	Primer sequence	Purpose
B5 3′ UTR F	atcccccgggATACCAGAAAGTCTCGTA	Cloning of 3′ UTR of *StBEL5*
B5 3′ UTR R	cagcccgggGCTAATCTAATAATGATA	into pIIIA/MS2-1 for library screening
B5 3′ UTR F w T3	AATTAACCCTCACTAAAGGGAGAATACCAGAAAGTCTCGTA	*In vitro* transcription of 3′ UTR of *StBEL5*
B5 D1 F	atcccccgggATACCAGAAAGTCTCGTA	Cloning of physical dissection (D1) of 3′ UTR
B5 D1 R	cagcccgggTATACTAGAAAGTGTGCTTT	of *StBEL5* into pIIIA/MS2-1 for validation
B5 T2 F	atcccccgggTATAGTATAGAAAAGAAGA	Cloning of physical dissection (T2) of 3′ UTR
B5 T2 R	cagcccgggTACTACTAGTTGTATCAATCT	of *StBEL5* into pIIIA/MS2-1 for validation
B5 UA F	atcccccgggTATTTTTTTTCTTTTGGGTTG	Cloning of physical dissection (UA-rich) of 3′ UTR
B5 UA R	cagcccgggTACAAAATATTTCAAATTATA	of *StBEL5* into pIIIA/MS2-1 for validation
B5 T1 F	atcccccgggACTCTTATATTGTG	Cloning of PT motif (T1) of 3′ UTR of *StBEL5*
B5 T1 R	atcccccgggAAAGAAGTATATAT	into pIIIA/MS2-1 for validation
B5 D1 F w T3	AATTAACCCTCACTAAAGGGAGAATACCAGAAAGTCTCGTA	*In vitro* transcription of D1 of *StBEL5*
B5 D2 F w T3	AATTAACCCTCACTAAAGGGAGATATACTTCTTTTTTTTATAG	*In vitro* transcription of D2 of *StBEL5*
B5 D3 F w T3	AATTAACCCTCACTAAAGGGAGATGGAAACTAGCTATAGTATA	*In vitro* transcription of D3 of *StBEL5*
B5 T1 F w T3	AATTAACCCTCACTAAAGGGAGAACTCTTATATTGTG	*In vitro* transcription of T1 of *StBEL5*
B5RBP2 F	tacggatccATGGCTTTCTTTAACAAAGC	Cloning of B5RBP2 into pET28a for
B5RBP2 R	cgatgtcgacGGCTCTTCTGTCTGCATAAT	expression of His-fusion protein
B5RBP3 F	tacggatccATGTCAAATTTTGATAGAGG	Cloning of B5RBP3 into pET28a for
B5RBP3 R	cgatgtcgacAGAAAATGGGTGAGAAGAAG	expression of His-fusion protein
B5RBP5 F	tacggatccATGGAGGACGACGTTGAGAT	Cloning of B5RBP5 into pET28a for
B5RBP5 R	cgatgtcgacGAAGTAGGGGCTGTAACGCA	expression of His-fusion protein
pGAD-C1 Seq	CGATGATGAAGATACCCCAC	Sequencing of the screened clones

*Note: Lower case letters represent restriction enzyme sites used for cloning*.
